# A Novel Thioredoxin-Dependent Peroxiredoxin (TPx-Q) Plays an Important Role in Defense Against Oxidative Stress and Is a Possible Drug Target in *Babesia microti*

**DOI:** 10.3389/fvets.2020.00076

**Published:** 2020-02-18

**Authors:** Houshuang Zhang, Zhonghua Wang, Jingwei Huang, Jie Cao, Yongzhi Zhou, Jinlin Zhou

**Affiliations:** Key Laboratory of Animal Parasitology of Ministry of Agriculture, Shanghai Veterinary Research Institute, Chinese Academy of Agricultural Sciences, Shanghai, China

**Keywords:** *Babesia microti*, thioredoxin, peroxidase-Q, antioxidant activity, drug target

## Abstract

Thioredoxin peroxidases (TPxs) are ubiquitous cysteine-based peroxidases that reduce peroxides as part of antioxidant defenses and redox signaling and are essential for *Babesia microti* protection against adverse environment agents like reactive oxygen species (ROS) and reactive nitrogen species (RNS). To better systematically understand TPxs, we identified a novel 2-Cys peroxiredoxin-Q (BmTPx-Q) of *B. microti*. The full-length BmTPx-Q gene is 653 bp that consists of an intact open reading frame of 594 bp that encodes a 197-amino acid protein. The predicted protein has a molecular weight of 22.3 kDa and an isoelectric point of 9.18. Moreover, BmTPx-Q showed low identity at the amino acid level to other peroxiredoxins (Prxs) among the currently known subfamilies. The recombinant BmTPx-Q protein (rBmTPx-Q) was expressed in *Escherichia coli* and purified with beads. The native protein BmTPx-Q was detected using mouse anti-BmTPx-Q polyclonal serum with western blotting and indirect immunofluorescence assay (IFA). In addition, enzyme activity was observed using nicotinamide adenine dinucleotide phosphate (NADPH) as substrate and triggered the NADPH-dependent reduction of the Trx/TrxR system. It was also discovered that BmTPx-Q mainly exists as a monomer whether under its native or functional states. In addition, when incubated with Chloroquine diphosphate salt for 24 h *in vitro*, the expression of BmTPx-Q showed a marked downward trend with the increase of drug concentration. These results suggest that *B. microti* uses BmTPx-Q to reduce and detoxify hydrogen peroxides to survive and proliferate inside the host. Furthermore, BmTPx-Q showed the lowest identity with host enzymes and could be a potential drug target for the development of novel strategies to control *B. microti* infection.

## Introduction

Babesiosis is a serious disease caused by infection with protozoan parasite *Babesia microti* that is transmitted to humans via the bite of an infected tick or a contaminated blood transfusion. There have been many reports of cases from Europe and the USA in recent years (1–3). Babesiosis has a huge impact on elderly, splenectomized or immunocompromised patients, leading to anemia, fatigue, and fever hematuria (4). Although babesiosis can be controlled by treatment with antiparasitic drugs, many drugs have safety issues (5). Therefore, identification of new drug targets is needed to develop novel therapeutic strategies and overcome setbacks, such as drug resistance.

It is well-known that *Babesia* has a complex life cycle, including both arthropod vectors and mammalian hosts, and that it replicates in the host's red blood cells. Since it is surrounded by oxygen-rich environments, the parasite is likely to counteract the toxic effects of reactive oxygen species (ROS) and reactive nitrogen species (RNS) that could induce oxidative DNA damage and lipid peroxidation (6, 7). The ROS and RNS are highly active compounds during normal cell metabolism. Therefore, to avoid the deleterious effects of ROS, various defense mechanisms have been adapted, such as non-enzymatic elements, which include glutathione (GSH) and vitamin C, and antioxidant enzymatic elements [i.e., catalase (CAT), superoxide dismutase (SOD), as well as peroxidase] (8). The parasite has developed a wide range of antioxidant systems, including peroxiredoxins (Prxs) (9), to keep their redox balance while living inside host erythrocytes (10, 11). Prxs have been a research topic of interest as a family of thiol-specific non-heme antioxidant peroxidases detoxifying hydrogen peroxide (H_2_O_2_), alkyl peroxides, and peroxynitrite (12). Moreover, Prxs are expressed at high levels in cells of almost all organisms, which protects cells against toxicity from ROS by reducing and detoxifying hydroperoxides, highlighting the importance of this protein family.

The Prx proteins have been divided into those that contain a single catalytic cysteine (Cys) residue and those that have an additional conserved residue (13). However, a new classification system has been suggested by Nelson and colleagues (14). Under this classification, Prxs are divided into six subfamilies based on the abundant sequence homology and structural similarity analyses, namely: Tpx, PrxQ-BCP, Prx1/AhpC, Prx5, Prx6, and AhpE (15–17). Alternatively, on the basis of the presence or relative locations of the resolving cysteine (Cr) residue, Prxs are classified into three types based on the distinct catalytic mechanisms: 1-Cys Prxs (Prx6 and AhpE), the typical 2-Cys Prx (Prx1/AhpC), and atypical 2-Cys (Tpx, TPx-Q, and Prx5) (18).

Prxs are collectively called thioredoxin peroxidases (TPxs) and constitute a large family of thiol-dependent peroxidases (18). Prxs are also potential drug targets. The studies demonstrated that these poorly cope with oxidative stress in Prx knockout strains of *Plasmodium falciparum* (19, 20). Recently, several TPxs of malaria parasites were identified, and the functional properties of the enzymes were considered key factors for the development of new drugs (21–24). Prx has also been reported to play key roles in innate immunity and inflammation (25) besides cellular redox signaling (26). Among these Prxs, the TPx-Q subfamily members have been proposed to be the most ancestral, most complex and the least systematically characterized (27). The TPx-Q proteins are thiol-based peroxidases, and are important for maintaining redox homeostasis in several organisms. TPx-Q is an atypical Prx that has already been identified in bacteria, parasites, and some lower eukaryotes but is not found in mammals (28–31).

TPx-Q, which is a cysteine-based peroxidase, has been detected in various protozoan parasites. TPx-Q-mediated resistance to major stresses largely relies on ROS degradation. TPx-Qs usually occurs at the monomeric state, with an intramolecular disulfide bond that are reduced by thioredoxins (Trx) (32). It has been shown that over-expression of TPx-Qs can cope with oxidative stresses in cyanobacteria. TPx-Q B from *Mycobacterium tuberculosis* is monomeric under reduced and oxidized states, and it is a thioredoxin-dependent and highly efficient fatty acid hydroperoxide reductase (33). Regardless of the mode of classification of these proteins, the catalytic mechanism of the enzyme remains central and relies on the redox-active cys, which is highly conserved in its amino acid sequence (34).

Although Prxs have been extensively studied, especially Tpx and Prx1 in *Plasmodium*, little is known about the Trx peroxidases-Q (TPx-Q) of *B. microti*. Sequencing of the full genome for *B. microti* has been completed (35), but the currently existing results are unilateral and unsystematic. In our previous study, we have done some basic research about the 2-Cys Trx peroxidases-1 and peroxidases-2 of *B. microti* (BmTPx-1 and BmTPx-2) (36, 37) and found evidence suggesting that the *B. microti* possesses at least two Prx subfamilies (Tpx and PrxQ). In this study, we identified and characterized a novel thioredoxin peroxidase (BmTPx-Q) from a strain of *B. microti*, analyzed the activity and assessed the BmTPx-Q expression after treatment with antiparasitic agents. All results suggest that *B. microti* can use BmTPx-Q to reduce and detoxify H_2_O_2_ for survival. Additionally, our new investigations on BmTPx-Q, a member of Prxs, provide new insights into the structure and function of Prx. We demonstrated that BmTPx-Q might act as an oxidative stress defensive molecule as well as drug target in *B. microti*.

## Materials and Methods

### Parasite Culture *in vivo*

The ATCC® PRA-99TM strain of *B. microti* was obtained from the American Type Culture Collection (ATCC, USA) and maintained by serial passage in BALB/C mice (SLAC Laboratory Animal Co., Ltd., China) using a method described previously (36). The parasites were isolated until the erythrocyte infection rate reached 30%−40%, which was confirmed with Giemsa-stained thin-blood smears.

### Parasite Culture and Treatment *in vitro*

For one independent experiment, *B. microti* was obtained from at least two mice; 3 days after infection (according to our protocol). The blood of the infected mice was carefully collected under aseptic conditions and added to bacteria-free anticoagulant. Then the blood was passed through a 27G needle three to five times. The blood cell debris was removed with a 5 μm syringe filter, and most of the parasites were isolated. Subsequently, the parasites were centrifuged at 2,000 rpm for 5 min with a horizontal centrifuge. The supernatant was discarded and the pellets were re-suspended with sterile culture medium. The pellets used for incubation experiments were then washed three times with phosphate-buffered saline (PBS, pH 7.2) containing 50 μg/mL gentamycin sulfate (Sigma) under aseptic conditions. Healthy red blood cells were taken from 21-days-old normal mice and were distributed in a 12-well culture plate. Subsequently, each well was cultured in 5% CO_2_ at 37°C in RPMI 1640 (Gibco, USA) supplemented with 1% penicillin/streptomycin (Gibco, USA) and 40% fetal bovine serum (Gibco, USA). The incubations were performed for 12 and 24 h at 37°C in a 5% CO_2_ incubator.

### Cloning and Sequence Analysis of *B. microti* TPx-Q (BmTPx-Q)

To find more novel genes from *B. microti*. Total RNA sequencing was performed to characterize all transcriptional activity. The brief method of preparing total RNA and cDNA for library construction and sequencing of the samples was described in our previous study (36). The full-length cDNA of the putative BmTPx-Q was generated by transcriptome analysis. The sequence was analyzed as described in our previous investigation (37). The Genetyx software (Software Development Co., Ltd., Tokyo, Japan) was used for the analysis of BmTPx-Q nucleotide and amino acid sequences. To further understand the relationships between BmTPx-Q and other TPx-Q genes, the inferred amino acid sequence of the BmTPx-Q was compared with other protein sequences (namely, the homologous TPx-Q proteins from different species) retrieved from the GenBank database using the National Center for Biotechnology Information (NCBI) Basic Local Alignment Search Tool (BLAST) (http://www.ncbi.nlm.nih.gov/blast/). The protein domain was identified using BLAST (http://www.ncbi.nlm.nih.gov/Structure/cdd/wrpsb.cgi). The signal peptide was predicted with the SignalP 4.1 server (http://www.cbs.dtu.dk/services/SignalP/).

### Expression and Purification of Recombinant BmTPx-Q (rBmTPx-Q)

The recombinant proteins (rBmTPx-Q) were produced as described previously (36). Briefly, the open reading frame (ORF) of BmTPx-Q was amplified by PCR with the following primer pair (forward) 5′-TT CAT ATG TTC AAA ATA CTG AAT TCA CGG-3′ and (reverse) 5′-TT CTCGAG CAG TTT ATC AAT AAA TTC-3′ (the underlined sequences contain the *Nde*I and *Xho*I restriction sites). The PCR product was inserted into the expression vector pET-30a (Novagen, USA). The recombinant plasmids harboring the BmTPx-Q (BmTPx-Q/pET-30a) coding sequence were transformed into *E. coli* (strain BL21). Induction of rBmTPx-Q Histidine-tag expression was performed using 1 mM isopropyl thio-b-D-galactoside (IPTG), followed by purification using Ni-NTA agarose beads (Merck-Millipore Corporation, USA). Purified rBmTPx-Q was evaluated a sample in 12% sodium dodecyl sulfate polyacrylamide gel electrophoresis (SDS–PAGE) using reducing conditions and stained it with Coomassie Brilliant blue R-250. Protein concentration was determined with the BCA assay (Thermo Fisher Scientific, USA).

### Antioxidant Activity Assay

To evaluate the antioxidant activity of rBmTPx-Q applying a mixed-function oxidation (MFO) assay (21, 29, 38). First, rBmTPx-Q (250, 500, and 1,000 μg/mL) was added to the reaction mixture, followed by incubation at 37°C for 1 h. Then, 500 ng of pBluescript SK(-) (Stratagene, USA) plasmid DNA was added to the reaction mixture, followed by incubation for an additional 2.5 h. Plasmid nicking was determined by using the MFO assay and analysis by 1% agarose gel electrophoresis, which was stained with DuRed dye (Fanbo Biochemicals Co. Ltd., China). Subsequently, to investigate whether rBmTPx-Q possesses peroxidase activity using a ferrithiocyanate system (39). The reaction mixtures (200 μL) containing rBmTPx-Q (1.5 μg protein) and 85 μL of buffer (0.5% glycerol/5 mM DTT/0.03 × PBS) was pre-incubated at room temperature (RT) for 2 min. H_2_O_2_ (1 mM) was used to start the reaction and was terminated with 40 μL of 26% trichloroacetic acid, which was added at 2-min intervals. The disappearance of H_2_O_2_ was monitored to assess rBmTPx-Q activity. In the reaction mixture, the remaining peroxide content was allowed to react with ~40 μL of 10 mM (NH_4_)_2_Fe(SO_4_)_2_ and 20 μL of 2.5 M KSCN, which formed a ferric thiocyanate complex that was red in color. The color intensity was measured at a wavelength of 475 nm using a microplate reader (SpectraMax M5; Molecular Devices, USA). In the presence of Trx and Trx reductase (TrxR), oxidation of nicotinamide adenine dinucleotide phosphate (NADPH) coupled with rBmTPx-Q to the reduction of H_2_O_2_ was examined using the method described by Kang et al. (40). NADPH oxidation was monitored in A_340_ in a 200-μL reaction mixture (0.14 μM TR, 6.4 μM Trx, 0.375 mM NADPH, 500 μg/mL rBmTPx-Q protein, 250 μM H_2_O_2_, 50 mM HEPES). The negative control (-)rBmTPx-Q indicates the absence of BmTPx-Q in reaction mixture.

### Western Blotting for the Native BmTPx-Q Protein

To identify the native BmTPx-Q protein in the lysate of *B. microti, B. microti*-infected red blood cells (iRBCs) were prepared from Kunming mice at 4, 5, 6, 7, and 8 days post-infection, and non-infected erythrocytes were used as a negative control. First, the infected blood samples were centrifuged in low speed and hemolyzed by red cell lysis buffer (Tiangen Biotech, China) after discarding the supernatant. The soluble fractions (20 mg per lane) were separated on a 12% SDS-PAGE. The Western blot analysis was performed as described previously (36). Briefly, the proteins were electrophoretically transferred to polyvinylidene difluoride (PVDF) membranes (Merck-Millipore Corporation, USA), and was blocked with 5% skim milk diluted in PBS/0.05% Tween (PBST) for 2 h at 37°C. Then, the membranes were incubated overnight at 4°C in mouse anti-rBmTPx-Q serum diluted to 1:200 in PBST. The blotted membranes were washed using PBST, followed by incubation in the presence of goat anti-mouse IgG antibody (horseradish peroxidase-conjugated; dilution, 1:2,000; Bethyl Laboratories, Inc., USA) for 1 h at 37°C. A washing step took place with PBST before applying an enhanced DAB chromogenic substrate kit (Tiangen Biotech, China) to visualize the bands in accordance with the manufacturer's guidelines.

### Expression Analysis of BmTPx-Q Post-infection

Assessment of BmTPx-Q expression post-infection was performed according to a method described elsewhere (41, 42). Briefly, we collected blood from mice infected with 1 × 10^8^ iRBCs at 1–10 days post-injection, from which total RNAs were extracted using TRIzol. The cDNA was constructed by a PrimeScript RT reagent kit with gDNA eraser (TaKaRa, Japan) and used in quantitative real-time PCR (qRT-PCR) analysis. The qRT-PCR was performed with SYBR® Premix Ex Taq TM II (TaKaRa, Japan) and a StepOne Plus PCR system (Applied Biosystems). The primer pair of BmTPx-Q were BmTPxQ-qRT-F: ACAAGCACAATCTCCCATAC (forward) and BmTPxQ-qRT-R: TCTCCAGCACTAACTCCC (reverse). The 18s ribosomal RNA of *B. microti* (Bm18S) (GenBank: XM_021481625.1) was used as an internal control. The primer pair of Bm18S were Bm18S-qRT-F: GTTATAGTTTATTTGATGTTCGTTT (forward) and Bm18S-qRT-R: AAGCCATGCGATTCGCTAAT (reverse). The parasitemia was calculated at each day post-infection. Analysis was performed using the 2^−ΔΔct^ method, and experimental values were expressed as relative amounts (43).

### Indirect Immunofluorescent Antibody Test

To determine the intracellular location of BmTPx-Q in *B. microti*, the test was performed as described previously (36). Briefly, thin smears of iRBCs were prepared and fixed in 50% acetone-50% methanol solution for 10 min at −30°C. Mouse antiserum against rBmTPx-Q was used as the primary antibody (dilution, 1:200), incubated at 37°C for 45 min. After it was washed with PBS, Alexa-Fluor® 488-conjugated goat anti-mouse IgG (Life Technologies Corporation, USA) was added as a secondary antibody (dilution, 1:2,000) and incubated at 37°C for 45 min. Then, the slides were washed using PBS and incubated with 0.5 μM 4′,6′-diamidino-2-phenylindole (Molecular Probes, Inc., USA) at RT for 20 min. The slides were later washed and mounted with Cytomation fluorescent mounting medium (Dako Corporation, USA) and visualized under a confocal laser-scanning microscope (Zeiss LSM 880, Germany).

### Transcript Changes of BmTPx-Q After Treatment With Antiparasitic Agents

To assess the mRNA relative expression profile of BmTPx-Q after treatment with antiparasitic agents, a short-term culture system of *B. microti* iRBCs was established *in vitro*. This study was performed as described in previous research (41). Briefly, *B. microti*-infected mice blood (~30% parasitemia) was collected and iRBCs were washed with PBS. A 12-well flat-bottom plate (Thermo, USA) was used for drug screening. The *B. microti* iRBCs (~2 × 10^7^) were cultured in RPMI 1640 (Life Technologies, USA) supplemented with 25 mM HEPES (Life Technologies, USA) and 40% fetal bovine serum (Life Technologies, USA) at 37°C in a 5% CO_2_ (44, 45). To evaluate the effect of drugs on BmTPx-Q gene expression, the iRBCs were grown *in vitro* for a short period before exposure to the three antiparasitic drugs (Quinine monohydrochloride dehydrate, Dihydroartemisinin, and Chloroquine diphosphate salt) (Sigma Aldrich, USA) was assessed. Quinine and Dihydroartemisinin were dissolved into dimethylsulfoxide (DMSO), while Chloroquine was dissolved in PBS. iRBCs were treated with various concentrations (20, 50, or 100 μM) of Quinine, Dihydroartemisinin, and Chloroquine at different timepoints (12, 24, and 36 h), meanwhile the controls were treated with DMSO or PBS. Relative BmTPx-Q transcript levels were assessed as previously described, by Reverse Transcription (RT)-PCR. Briefly, total RNA was isolated from infected RBCs using TRI solution (Life Technologies Corporation, USA), and RT-PCR was carried out applying 1 μg of total RNA and specific primers by a PrimeScript™ One-Step RT-PCR Kit (Takara, China). Conditions for the PCRs were as follows: 95°C for 30 s; 95°C for 5 s, 60°C for 35 s, for 40 cycles. The experiment was repeated in triplicate.

### Statistical Analysis

A GraphPad PRISM 5 software (GraphPad Software Inc., CA, USA) was used for the data analysis. The mean ± standard deviation (SD) of each group was calculated. The differences between groups were assessed using two-tailed *t*-tests. *P* < 0.05 was considered significant and *P* < 0.01 was considered highly significant.

## Results

### Identification and Characterization of the BmTPx-Q Gene

**Figure 1:**
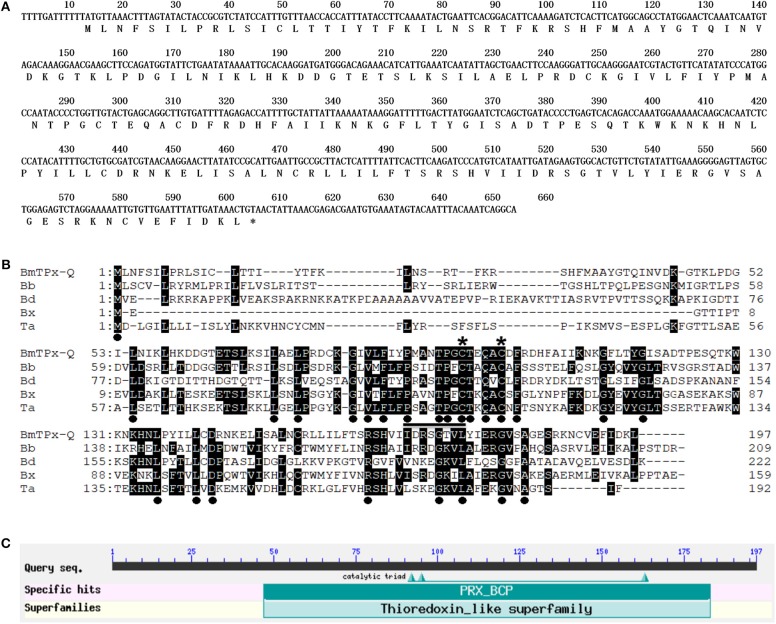
Analysis of the sequence and primary structure of BmTPx-Q. **(A)** Nucleotide and deduced amino acid sequences of BmTPx-Q. **(B)** BmTPx-Q multiple sequence alignment analysis. *B. microti* (BmTPx-Q); *Babesia bigemina* (Bb: XP_012767998.1); *Blastomyces dermatitidis* (Bd: EEQ83458.1); *Babesia* sp. *Xinjiang* (Bx: XP_028870106.1); *Theileria annulata* (Ta: XP_954500.1). The dot indicates the identical amino acids in all sequences, an asterisk indicates the conserved cysteine residue, and the conserved PxxxTxxC-motif around the active site is underlined. **(C)** Analysis of the active domains in the BmTPx-Q amino acid sequence as identified by NCBI blast.

The identified full-length cDNA of BmTPx-Q has 653 bp in which includes a single ORF of 594 bp. Sequence analysis indicated that the ORF of BmTPx-Q gene encodes a protein of 197 amino acids with a theoretical molecular weight and isoelectric point of 22.3 kDa and 9.18, respectively. The amino acid sequence was deduced from the cDNA sequence of BmTPx-Q (Figure 1A). SignalP server indicated that BmTPx-Q has a signal peptide. The BmTPx-Q protein sequence was 98% identical to that of *B. microti* Prx Q (RI strain, XP_012647890.1), showing 35% sequence similarity with Prx Q of *Babesia bigemina* (XP_012767998.1), 35% with Prx Q of *Blastomyces dermatitidis* (EEQ83458.1), 39% with Prx Q of *Babesia* sp. *Xinjiang* (XP_028870106.1), and 43% with Ahp/TSA family-related protein, putative of *Theileria annulata* (XP_954500.1) (Figure 1B). The sequence alignment showed that the conserved peroxidatic cysteine (Cp) of BmTPx-Q is located at position 95 in a PxxxTxxC-motif, and an additional conserved cysteine at position 100 in BmTPx-Q (Figure 1B). The BmTPx-Q sequence contains a Thioredoxin-dependent hydroperoxide peroxidase activity of bacterioferritin comigratory protein (PRX-BCP) domain, the PRX-BCP is a new member of the thiol-specific antioxidant protein (TSA)/Alkyl hydroperoxide peroxidase C (AhpC) family (Figure 1C). The BmTPx-Q belonging to the Thioredoxin-like superfamily, it also contains a catalytic triad on conserved domain PRX_BCP, 3 of 3 of the residues that compose this conserved feature (Figure 1C).

### Expression and Purification of the Recombinant BmTPx-Q

**Figure 2:**
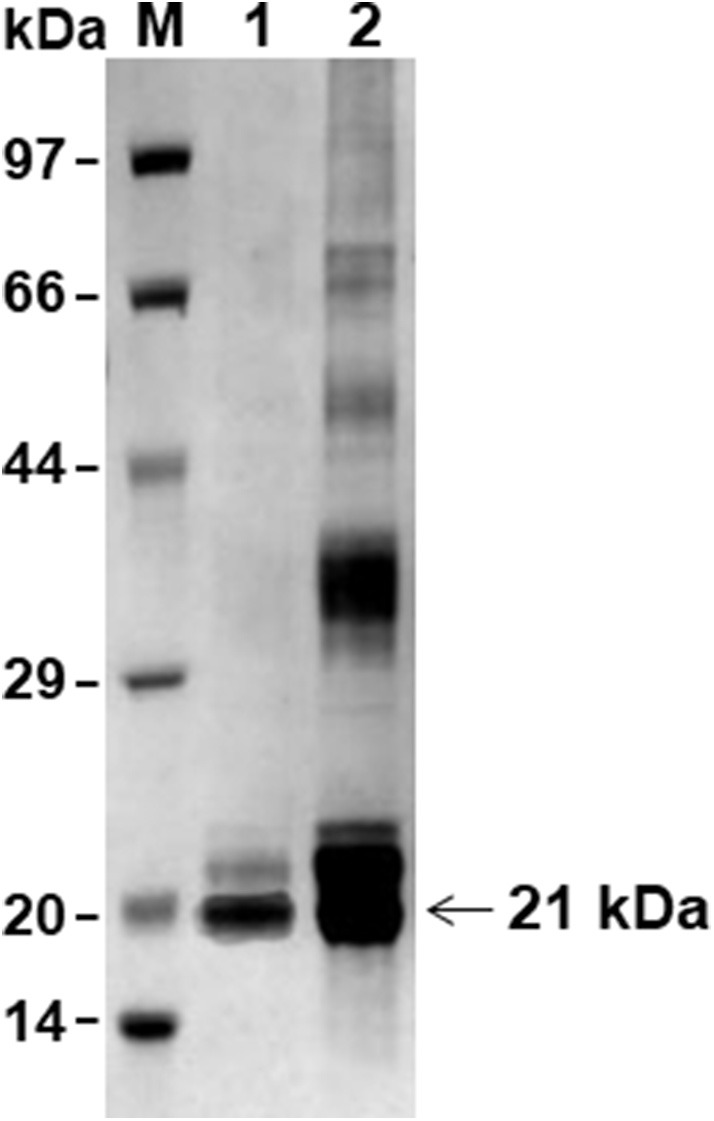
SDS-PAGE analysis of the rBmTPx-Q-His fusion protein. Analysis of expression and purification of rBmTPx-Q by reducing SDS-PAGE (12%). Lane M: standard protein molecular weight marker; Lane 1: purified rBmTPx-Q; Lane 2: induced cell lysates. Purified rBmTPx-Q-His was ~21 kDa and was used in subsequent antibody production and antioxidant activity assays.

The PCR product was cloned into the pET30a vector and the recombinant protein was successfully expressed in *E. coli* BL21 (DE3) as a his-tagged protein. The rBmTPx-Q protein was purified with Ni-NTA agarose beads and was analyzed by SDS/PAGE. As shown in Figure 2, the purified protein showed a single band (~21 kDa) by 12% SDS/PAGE in the presence of DTT (reducing).

### Antioxidant Activity of rBmTPx-Q

**Figure 3:**
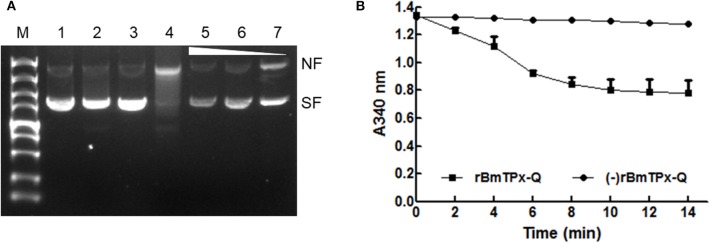
Antioxidant activity of the BmTPx-Q protein. **(A)** The pBluescript plasmid DNA was incubated with reaction mixture including the rBmTPx-Q, and nicking of the supercoiled plasmids by MFO was evaluated on 1% agarose gels stained with ethidium bromide. M, DNA marker; 1, DNA + H_2_O; 2, DNA + DTT; 3, 180 ng DNA + FeCl_3_; 4, DNA + MFO; 5, 1,000 μg/mL rBmTPx-Q + MFO + DNA; 6, 500 μg/mL rBmTPx-Q + MFO + DNA; and 7, 250 μg/mL rBmTPx-Q + MFO + DNA. The nicked form (NF) and supercoiled form (SF) of the plasmids are indicated on the right, and the triangle shows the increasing concentration of the recombinant proteins. **(B)** Peroxidase activity of rBmTPx-Q. NADPH oxidation coupled by rBmTPx-Q (solid line) to reduction of H_2_O_2_ in the presence of the *E. coli* Trx/Trx R system. NADPH oxidation was monitored as the decrease in A 340 in a 200-μL reaction mixture (0.14 μM TR, 6.4 μM Trx, 0.375 mM NADPH, 500 μg/mL rBmTPx-Q, 250 μM H_2_O_2_, 50 mM HEPES). The dotted line indicates the absence of rBmTPx-Q in reaction mixture. Data are presented as the means of three independent experiments.

The activity was evaluated using the MFO assay (Figure 3A). In this assay, FeCl_3_ and DTT generate hydroxyl radicals in the reaction mixture induced nicks in the supercoiled plasmid DNA, thereby altering the mobility of DNA during agarose electrophoresis. Both FeCl_3_ and DTT generated nicks in the DNA in the absence of rBmTPx-Q. Thus, there was an apparent increase in DNA size (Figure 3A, lane 4). However, nicks in the plasmid DNA were detected after purified rBmTPx-Q was added to the reaction mixtures at 1,000, 500, and 250 μg/mL concentrations (Figure 3A, lanes 5–7). The results indicate rBmTPx-Q has antioxidant activity. TPx-Q proteins are thiol-based peroxidases that catalyze the reduction of H_2_O_2_. The ability of the rBmTPx-Q to remove H_2_O_2_ was evaluated utilizing a ferrithiocyanate system. The rBmTPx-Q was examined by monitoring oxidation of NADPH in the *E. coli* Trx/TrxR system in the presence of DTT. As demonstrated in Figure 3B, rBmTPx-Q showed peroxidase activity in the presence of the Trx system (Trx, TR, and NADPH) with a concentration-dependent manner. These results indicate that the atypical 2-Cys TPx-Q peroxidases use thioredoxin as a reductant. The results showed a decrease at A_340_ nm in the presence of rBmTPx-Q, possibly due to the oxidation of NADPH after the addition of H_2_O_2_. Within 10 min, 1.5 μg of rBmTPx-Q destroyed nearly about half of 1 mM H_2_O_2_. This result suggested that rBmTPx-Q can be described as a thioredoxin peroxidase.

### Expression Analysis of BmTPx-Q in *B. microti* Post-infection

**Figure 4:**
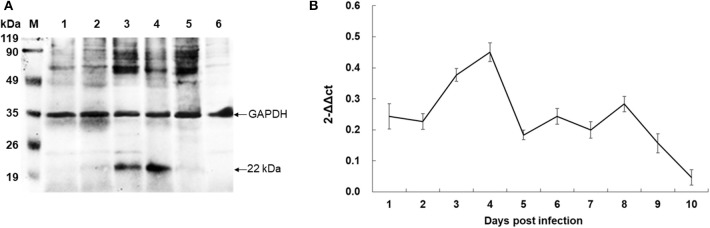
Expression analysis of BmTPx-Q in *B. microti* post-infection. **(A)** Western blot analysis of the native BmTPx-Q. Lane M: standard protein molecular weight marker; Lanes 1–5: *B. microti* infected mouse erythrocyte lysates on 5th, 6th, 7th, 8th, and 9th day post-infection; Lane 6: uninfected mouse erythrocyte lysate; mouse anti-rBmTPx-Q serum was used as primary antibody in this Western blot analysis and revealed a band of ~22 kDa. **(B)** Relative expression analysis of BmTPx-Q at different days post-infection. The x-axis refers to the day of infection of mouse RBCs with *B. microti*; the y-axis refers to the relative BmTPx-Q expression level in mouse RBCs on different days post-infection by using the 2^−ΔΔCT^ mean.

To identify the native BmTPx-Q protein in *B. microti*, western blot analysis was performed using the mouse anti-rBmTPx-Q serum. The results showed that an ~22 kDa band was detected in the iRBC lysates 7 and 8 days post-infection (Figure 4A, lines 3 and 4), which revealed the predicted monomeric size, whereas no specific bands were detected in the non-infected control (Figure 4A, line 6). The size of the recombinant BmTPx-Q band was similar to that of the native BmTPx-Q. The results suggested that BmTPx-Q protein has high immunogenicity and can induce the host's immune system and also BmTPx-Q exists primarily in a monomeric form without the formation of intermolecular disulfide bonds. Moreover, total RNA of iRBCs from different days post-infection was used to investigate the expression profile of BmTPx-Q by qRT-PCR analysis. BmTPx-Q expression peaked twice on 4 and 8 days post-infection and declined suddenly on 5 and 9 days post-infection (Figure 4B). Blood smears were stained with Giemsa to assess the infection by calculating the ratio of iRBCs. *B. microti* was detected in iRBCs on the first day post-infection. The parasitemia ratio increased until 5 days post-infection, and started to decline on 6 days post-infection until reaching undetectable levels by day 10 post-infection (Figure S1).

### Immunofluorescence Assays

**Figure 5:**
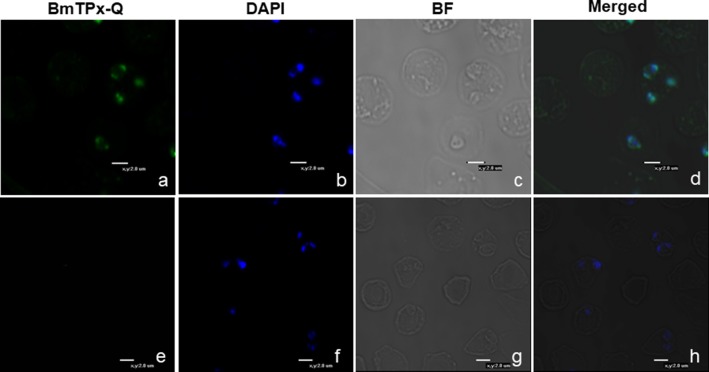
Immunofluorescence microscopy analysis of the cellular localization of TPx-Q in *B. microti*-infected RBCs. Antiserum against BmTPx-Q **(a–d)** or normal mouse serum **(e–h)** was incubated with acetone-fixed *B. microti*-infected mice erythrocytes and stained with Alexa Fluor 488-conjugated goat anti-mouse IgG antibody. **(a,e)** Incubation with anti-rBmTPx-Q or normal mouse serum staining; **(b,f)** DAPI staining; **(c,g)** brightfield; and **(d,h)** merged images. Scale bar indicates 2.0 μm.

A thin blood smear of *B. microti*-infected RBCs (~20% parasitemia) was used by indirect immunofluorescence assay (IFA) with the mouse anti-rBmTPx-Q serum. The blue fluorescence indicates the nucleus of *B. microti*, whereas the green fluorescence shows BmTPx-Q located within the nucleus of *B. microti* merozoites in iRBCs (Figure 5a). Additionally, co-localization of anti-BmTPx-Q signal (green) with DAPI (blue) indicates that most of BmTPx-Q is present in the cytoplasm of the parasites (Figure 5d, Merged). In the control sample, no green fluorescence was detected in the iRBCs which were incubated with serum collected from uninfected mice (Figure 5e). Therefore, our results show that BmTPx-Q is expressed in *B. microti* merozoite cytoplasm.

### Transcript Analysis of BmTPx-Q After Treatment With Antiparasitic Agents

**Figure 6:**
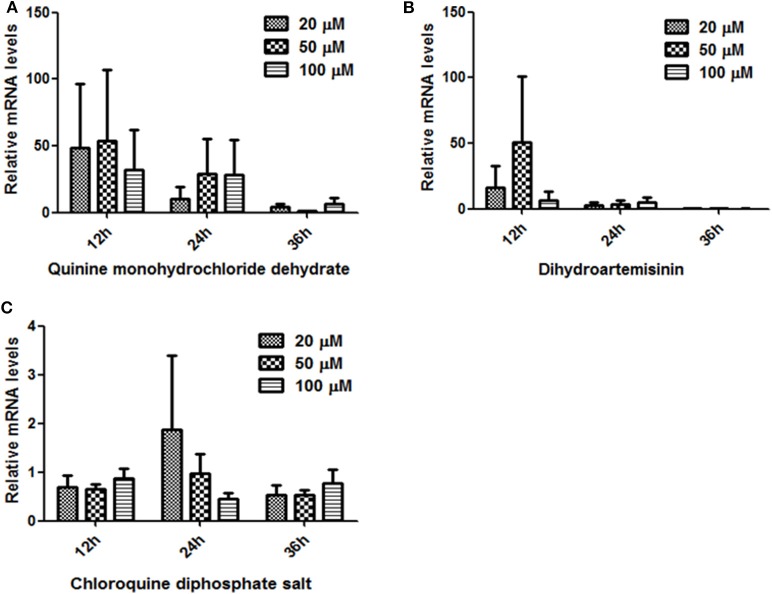
Relative expression analysis of BmTrx-Q in iRBCs exposed to anti-parasitic agents for 12, 24, and 36 h. The iRBCs were treated with different concentrations (20, 50, 100 μM) of Quinine **(A)**, Dihydroartemisinin **(B)**, and Chloroquine **(C)**.

The RT-PCR was conducted using various cDNA templates isolated samples treated with different doses of Quinine monohydrochloride dehydrate, Dihydroartemisinin, or Chloroquine at 12, 24, and 36 h. As shown in Figures 6A,B, BmTPx-Q was expressed in three different time points, although there was no significant difference, but a decreasing trend was observed as time went by in the three different concentrations under Quinine and Dihydroartemisninin compared to the control group (we have subtracted the control group data when analyzing). In addition, BmTPx-Q was expressed at the highest level in 20 and 50 μM at 24 h under Chloroquine diphosphate salt (Figure 6C). High BmTPx-Q expression in the parasites was due to adverse environmental factors, which increases gradually to peak at 24 h before decreasing sharply in the subsequent time points. The growth status of *B. microti* after that was severely inhibited at 36 h, which was also confirmed through Giemsa-stained thin-blood films (Figure S2). That can explain the low expression level of BmTPx-Q in the subsequent stages, also indicating that *B. microti* might be inhibited by these three drugs. These findings suggest that BmTPx-Q might be implicated by different mechanisms in the response of *B. microti* to Quinine, Dihydroartemisninin, and Chloroquine. Although Prxs play a role in protection against oxidative damage in parasites and ensure a certain degree of defense, the effects of external factors can be irreversible.

## Discussion

Prxs reduce peroxides using a peroxidatic cysteine residue, and the Cys are essential for enzyme activity. In atypical 2-Cys Prxs, which are also named Type II Prxs, the position of one of two cysteine residues is not conserved (8, 46, 47). The typical 2-Cys Prxs have physiological functions as peroxidase, 1-Cys Prx and atypical 2-Cys act as monomers, whereas typical 2-Cys Prxs act as dimers (48, 49).

To better systematically understand TPxs, we previously have identified the Prxs (BmTPx-1 and BmTPx-2) from *B. microti* (36, 37). Herein, we extended these efforts to select additional members of this group. A BLAST search revealed that the BmTPx-Q has low sequence similarities with those of Prxs of host species. This may indicate that this protein may have a good prospect in terms of drug targets. As expected, like all 2-Cys-containing Prxs, BmTPx-Q possesses the PxxxTxxC-motif and beside two conserved cysteine residues Cp and Cr that are essential for peroxidase activity. The Cp is located at the N-terminal end of the protein and Cr is usually four residues or 30 amino acids away from Cp (50). In our study, BmTPx-Q as a 2-Cys Prx, Cys95 being Cp, which is essential for peroxidase activity, whereas Cys100 acts as Cr. For 2-Cys Prxs, the Cp is oxidated by a peroxide substrate to generate a Cys sulfenic acid (Cys-SOH) intermediate, which reacts with the Cr to form an monomer or dimmer (18, 51). The disulfide bond is reduced to reform the reduced Cp by an external reducing substrate of Trx/TrxR system with an atypical 2-Cys catalytic mechanism (32). Afterwards, the generated TPx-Q is ready for another catalytic cycle. Su et al. (30) indicated that the two cysteine residues are essential for enzymatic activity by mutation analysis. Furthermore, they proved that *C. glutamicum* TPx-Q catalytically eliminates peroxides by exclusively receiving electrons from Trx/TrxR system (30). Unlike the typical 2-Cys Prx proteins, TPx-Q may exist as monomers, dimers, or a mixture (52). In this study, BmTPx-Q may exist mainly in monomeric form with an intramolecular disulfide bond. Moreover, it has been documented that the three forms of TPx-Q contain a wealth of information useful to the field (27).

To assess the enzymatic activity and generate specific antibodies against BmTPx-Q, the recombinant proteins were expressed as a his-tagged protein in *E. coli* purified by agarose beads (Figure 2). It is now known that Prxs are prevalent in prokaryotic and eukaryotic organisms and are essential against various oxidative stresses through catalyzing the reduction of H_2_O_2_ and organic hydroperoxides to keep the cellular redox balance (53). In addition, Prxs are more than just simple peroxide-eliminating enzymes. They are localized to various subcellular compartments and function as regulators of local H_2_O_2_ levels. The studies of transcription revealed that the Prx Q has a function in oxidant defense (54). Therefore, the antioxidant activities of rBmTPx-Q were evaluated by the MFO assay (Figure 3A). In MFO assay, FeCl_3_ and DTT generate hydroxyl radicals that produced nicks in the supercoiled DNA in the reaction mixture, which could be monitored by changing the running behavior of the DNA in electrophoresis (38). Therefore, the presence of rBmTPx-Q in the reaction mixtures with 250, 500, and 1,000 μg/mL concentration prevented the damage of DNA (Figure 3A), suggesting the antioxidant activities of rBmTPx-Q. Data shown in Figure 3B demonstrated that the peroxidase activity of rBmTPx-Q acts as an antioxidant enzyme, the results were consistent that Prx as a peroxidase to reduce H_2_O_2_ via the parasitic Trx system in previous study (55).

Although Prxs are primarily as peroxidases, these also function as chaperones or phospholipases and are involved in redox signaling (48, 56). The chaperone or the phospholipase activity of Prxs is independent with the catalytic cysteine residues. Molecular chaperones can assist the covalent folding of proteins and prevent the protein aggregation (57). Su et al. (30) found that TPx-Q functions as molecular chaperone and peroxidase. Cho et al. (58) first reported that Prx Q of *Deinococcus radiodurans* R1 is a monomeric atypical 2-Cys Prx and has dual activity as peroxidase and a molecular chaperone. However, the molecular chaperones function of BmTPx-Q needs to be further studied.

Meanwhile, we collected the *B. microti* infected mouse erythrocyte lysates post-infection and detected the native BmTPx-Q protein in the lysate of *B. microti* using the mouse anti-rBmTPx-Q serum. Western blotting showed an ~22 kDa band in the iRBC lysates 7 and 8 days post-infection (Figure 4A, lines 3 and 4). This indicates that the native BmTPx-Q was expressed in large quantities that could be detected on the 7th and 8th day after infection. Moreover, the expression of BmTPx-Q peaked at both 4 and 8 days post-infection by qRT-PCR analysis (Figure 4B). Based on the growth curve of parasitemia, *B. microti* was undergoing a rapid propagation process in RBCs 4 days post-infection. During the erythrocytic stages, *B. microti* are exposed to oxygen-rich environments and must secrete large amounts of antioxidants, such as Trx or Prx to act against oxidative stress and protect the parasites (23). Furthermore, the native *B. microti* Prx protects DNA from oxidative damage (23, 59). From a rational point of view, high expression levels of BmTPx-Q at 4 days post-infection helped *B. microti* to cope with the oxidative stress during the rapid propagation process. The level of BmTPx-Q expression reached a peak, whereas *B. microti* parasitemia decreased to low levels at 8 days post-infection, similar results were also observed for *B. microti* other antioxidant enzymes, such as peroxiredoxin 2, thioredoxin 2, and thioredoxin 3 in previous studies (37, 41, 42). The molecular immune mechanisms of high expression levels of these antioxidant molecules need to be further investigated.

In addition, previous research has reported that Prxs could induce protective immunity against *Leishmania* major infections and microfilaria *Brugiamalayi* infections in mice and *Fasciola hepatica* infections in goats (60). Since Prxs have potential as candidate vaccines for parasite species, the enzymatic activity may be related to the protective efficacy of these antigens. Our laboratory will evaluate the potential of this vaccine candidate in future studies.

IFA remains the most widely used method to localize proteins in organisms' intact cells. Figure 5 shows the expression patterns of BmTPx-Q in the red blood cells. The green fluorescence surrounding merozoite nuclei indicates enzyme expression in the cytoplasm of the parasite (Figure 5a). Based on a previous report, 1-Cys-Prx is also upregulated in the cytoplasm at the trophozoite stage, which is the metabolically active phase of *P. falciparum* (61). Unlike thioredoxin peroxidase-1 (TPx-1) of *B. microti*, BmTPx-Q exhibits a dot-like pattern expression in the parasite. Further investigation into whether BmTPx-Q and BmTPx-1 or BmTPx-2 co-exist in the peroxidase active organelles is required.

There was no significant difference regarding the mRNA relative expression profile of BmTPx-Q in the groups treated with the antiparasitic agents, compared to the control group, but a decreasing trends were observed against time (Figure 6). Three drugs managed to inhibit *B. microti* growth, but there was no marked effect on BmTPx-Q expression or activity. It is necessary to find specific inhibitors or drugs able to target BmTPx-Q, which will further confirm the importance of this molecule for *B. microti* survival. Moreover, it is necessary to confirm the TPx-Q whether as an oxidative stress defensive molecule as well as drug target in other strain or species of *Babesia*.

## Conclusions

We characterized a BmTPx-Q in *B. microti*, and our results suggest that TPx-Q acts as a thioredoxin-dependent monomeric peroxidase that contributes to the resistance against oxidative in *B. microti*. BmTPx-Q is a monomeric form with two Cys that form an intramolecular disulfide bond. Since BmTPx-Q has an antioxidant activity, apparently it has a crucial role in the reduction of ROS. Concerning other aspects, *Babesia* parasites have other antioxidant proteins, such as SOD and Gpx. Thus, it is necessary to study the correlations between BmTPx-Q and these antioxidant proteins. Our data on BmTPx-Q can be used to investigate the precise role and biological functions of BmTPx-Q in the parasite. These findings may be relevant to the field of parasitological research and lead to the development of an anti-Babesiosis drug.

## Data Availability Statement

All datasets generated for this study are included in the article/Supplementary Material.

## Ethics Statement

The Institutional Animal Care and Use Committee of the Shanghai Veterinary Research Institute (IACUC Approval Number SHVRI-mo-2017070806) approved and the Animal Ethical Committee of Shanghai Veterinary Research Institute authorized this investigation.

## Author Contributions

HZ and JZ conceived and designed this study. HZ and ZW performed the experiments. JH conducted the molecular analysis. JC performed the serology. YZ conducted the statistical analysis. All authors have read and approved the final version of this manuscript.

### Conflict of Interest

The authors declare that the research was conducted in the absence of any commercial or financial relationships that could be construed as a potential conflict of interest.

## Supplementary Material

Figure S1 Giemsa-stained thin-blood film of the parasitemia. D0: Uninfected mouse RBCs; D1–D10: Days post-infection with *B. microti* of mouse RBCs. Scale bar indicates 5.0 μm.

Figure S2 The growth status of *B. microti* after treatment with antiparasitic agents at 36 h. iRBCs were treated with various concentrations (20, 50, or 100 μM) of Quinine, Dihydroartemisinin, and Chloroquine at 36 h, the controls were treated with DMSO. Thin-blood film was stained with Giemsa. Scale bar indicates 10.0 μm.

## References

[1] Herwaldt BL Caccio S Gherlinzoni F Aspöck H Slemenda SB Piccaluga P. Molecular characterization of a non-*Babesia divergens* organism causing zoonotic babesiosis in Europe. Emerg Infect Dis. (2003) 9:942–8. 10.3201/eid0908.020748 12967491 PMC3020600

[2] Obiegala A Pfeffer M Pfister K Karnath C Silaghi C. Molecular examinations of *Babesia microti* in rodents and rodent-attached ticks from urban and sylvatic habitats in Germany. Ticks Tick Borne Dis. (2015) 6:445–9. 10.1016/j.ttbdis.2015.03.005 25922232

[3] Vannier E Krause PJ. Human babesiosis. N Engl J Med. (2012) 366:2397–407. 10.1056/NEJMra1202018 22716978

[4] Homer MJ Aguilar-Delfin I Telford SR III Krause PJ Persing DH. Babesiosis. Clin Microbiol Rev. (2000) 13:451–69. 10.1128/CMR.13.3.451 10885987 PMC88943

[5] Vial HJ Gorenflot A. Chemotherapy against babesiosis. Vet Parasitol. (2006) 138:147–60. 10.1016/j.vetpar.2006.01.048 16504402

[6] Gray J Zintl A Hildebrandt A Hunfeld KP Weiss L. Zoonotic babesiosis: overview of the disease and novel aspects of pathogen identity. Ticks Tick Borne Dis. (2010) 1:3–10. 10.1016/j.ttbdis.2009.11.003 21771506

[7] Robinson MW Hutchinson AT Dalton JP Donnelly S. Peroxiredoxin: acentral player in immune modulation. Parasite Immunol. (2010) 32:305–13. 10.1111/j.1365-3024.2010.01201.x 20500659

[8] Reuter S Gupta SC Chaturvedi MM Aggarwal BB. Oxidative stress, inflammation, and cancer: how are they linked? Free Radic Biol Med. (2010) 49:1603–16. 10.1016/j.freeradbiomed.2010.09.006 20840865 PMC2990475

[9] Kawazu S Komaki-Yasuda K Oku H Kano S. Peroxiredoxins in malaria parasites: parasitologic aspects. Parasitol Int. (2008) 57:1–7. 10.1016/j.parint.2007.08.001 17890140

[10] Bosch SS Kronenberger T Meissner KA Zimbres FM Stegehake D Izui NM. Oxidative stress control by apicomplexan parasites. Biomed Res Int. (2015) 2015:351289. 10.1155/2015/351289 25722976 PMC4324108

[11] Poole LB Hall A Nelson KJ. Overview of peroxiredoxins in oxidant defense and redox regulation. Curr Protoc Toxicol. (2011) Chapter 7:Unit7.9. 10.1002/0471140856.tx0709s49 21818754 PMC3156475

[12] Fujii J Ikeda Y. Advances in our understanding of peroxiredoxin, a multifunctional, mammalian redox protein. Redox Rep. (2002) 7:123–30. 10.1179/135100002125000352 12189041

[13] Nickel C Trujillo M Rahlfs S Deponte M Radi R Becker K. *Plasmodium falciparum* 2-Cys peroxiredoxin reacts with plasmoredoxin and peroxynitrite. Biol Chem. (2005) 386:1129–36. 10.1515/BC.2005.129 16307478

[14] Nelson KJ Knutson ST Soito L Klomsiri C Poole LB Fetrow JS. Analysis of the peroxiredoxin family: using active-site structure and sequence information for global classification and residue analysis. Proteins. (2011) 79:947–64. 10.1002/prot.22936 21287625 PMC3065352

[15] Chae HZ Robison K Poole LB Church G Storz G Rhee SG. Cloning and sequencing of thiol specific antioxidant from mammalian brain: alkyl hydroperoxide reductase and thiol-specific antioxidant define a large family of antioxidant enzymes. Proc Natl Acad Sci USA. (1994) 91:7017–21. 10.1073/pnas.91.15.7017 8041738 PMC44329

[16] Karplus PA. A primer on peroxiredoxin biochemistry. Free Radic Biol Med. (2015) 80:183–90. 10.1016/j.freeradbiomed.2014.10.009 25452140 PMC4355262

[17] Perkins A Nelson KJ Parsonage D Poole LB Karplus PA. Peroxiredoxins: guardians against oxidative stress and modulators of peroxide signaling. Trends Biochem Sci. (2015) 40:435–45. 10.1016/j.tibs.2015.05.001 26067716 PMC4509974

[18] Hall A Karplus PA Poole LB. Typical 2-Cys peroxiredoxins-structures, mechanisms and functions. FEBS J. (2009) 276:2469–77. 10.1111/j.1742-4658.2009.06985.x 19476488 PMC2747500

[19] Krnajski Z Gilberger TW Walter RD Cowman AF Müller S. Thioredoxin reductase is essential for the survival of *Plasmodium falciparum* erythrocytic stages. J Biol Chem. (2002) 277:25970–5. 10.1074/jbc.M203539200 12004069

[20] Sarma GN Nickel C Rahlfs S Fischer M Becker K Karplus PA. Crystal structure of a novel *Plasmodium falciparum* 1-Cys peroxiredoxin. J Mol Biol. (2005) 346:1021–34. 10.1016/j.jmb.2004.12.022 15701514

[21] Hakimi H Asada M Angeles JM Inoue N Kawazu S. Cloning and characterization of *Plasmodium vivax* thioredoxin peroxidase-1. Parasitol Res. (2012) 111:525–9. 10.1007/s00436-012-2864-3 22392134

[22] Hakimi H Suganuma K Usui M Masuda-Suganuma H Angeles JM Asada M. *Plasmodium knowlesi* thioredoxin peroxidase 1 binds to nucleic acids and has RNA chaperone activity. Parasitol Res. (2014) 113:3957–62. 10.1007/s00436-014-4060-0 25092384

[23] Hakimi H Goto Y Suganuma K Angeles JM Kawai S Inoue N. Development of monoclonal antibodies against *Plasmodium falciparum* thioredoxin peroxidase 1 and its possible application for malaria diagnosis. Exp Parasitol. (2015) 154:62–6. 10.1016/j.exppara.2015.04.018 25913091

[24] Usui M Masuda-Suganuma H Fukumoto S Angeles JM Hakimi H Inoue N. Effect of thioredoxin peroxidase-1 gene disruption on the liver stages of the rodent malaria parasite *Plasmodium berghei*. Parasitol Int. (2015) 64:290–4. 10.1016/j.parint.2014.09.013 25284813

[25] Knoops B Argyropoulou V Becker S Ferté L Kuznetsova O. Multiple roles of peroxiredoxins in inflammation. Mol Cells. (2016) 39:60–4. 10.14348/molcells.2016.2341 26813661 PMC4749876

[26] Hampton MB O'Connor KM. Peroxiredoxins and the regulation of cell death. Mol Cells. (2016) 39:72–6. 10.14348/molcells.2016.2351 26810076 PMC4749878

[27] Perkins A Gretes MC Nelson KJ Poole LB Karplus PA. Mapping the active site helix-to-strand conversion of CxxxxC peroxiredoxin Q enzymes. Biochemistry. (2012) 51:7638–50. 10.1021/bi301017s 22928725 PMC3549014

[28] Buchko GW Perkins A Parsonage D Poole LB Karplus PA. Backbone chemical shift assignments for *Xanthomonas campestris* peroxiredoxin Q in the reduced and oxidized states: a dramatic change in backbone dynamics. Biomol NMR Assign. (2016) 10:57–61. 10.1007/s12104-015-9637-8 26438558 PMC4789116

[29] Masatani T Asada M Ichikawa-Seki M Usui M Terkawi MA Hayashi K. Cloning and characterization of a 2-Cys peroxiredoxin from *Babesia gibsoni*. J Vet Med Sci. (2014) 76:139–43. 10.1292/jvms.13-0274 24025459 PMC3979947

[30] Su T Si M Zhao Y Liu Y Yao S Che C. A thioredoxin-dependent peroxiredoxin Q from *Corynebacterium glutamicum* plays an important role in defense against oxidative stress. PLoS ONE. (2018) 13:e0192674. 10.1371/journal.pone.0192674 29438446 PMC5811025

[31] Tailor V Ballal A. Novel molecular insights into the function and the antioxidative stress response of a Peroxiredoxin Q protein from cyanobacteria. Free Radic Biol Med. (2017) 106:278–87. 10.1016/j.freeradbiomed.2017.01.031 28159708

[32] Kong W Shiota S Shi Y Nakayama H Nakayama K. A novel peroxiredoxin of the plant *Sedum lineare* is a homologue of *Escherichia coli* bacterioferritin co-migratory protein (Bcp). Biochem J. (2000) 351:107–14. 10.1042/bj3510107 10998352 PMC1221340

[33] Reyes AM Vazquez DS Zeida A Hugo M Piñeyro MD De Armas MI TPx-Q B from *Mycobacterium tuberculosis* is a monomeric, thioredoxin-dependent and highly efficient fatty acid hydroperoxide reductase. Free Radic Biol Med. (2016) 101:249–60. 10.1016/j.freeradbiomed.2016.10.005 27751911

[34] Rhee SG Kil IS. Multiple functions and regulation of mammalian peroxiredoxins. Annu Rev Biochem. (2017) 86:749–75. 10.1146/annurev-biochem-060815-014431 28226215

[35] Cornillot E Kaddour KH Dassouli A Noel B Ranwez V Vacherie B. Sequencing of the smallest Apicomplexan genome from the human pathogen *Babesia microti*. Nucleic Acids Res. (2012) 40:9102–14. 10.1093/nar/gks700 22833609 PMC3467087

[36] Zhang H Wang Z Gong H Cao J Zhou Y Zhou J. Identification and functional study of a novel 2-cys peroxiredoxin (BmTPx-1) of *Babesia microti*. Exp Parasitol. (2016) 170:21–7. 10.1016/j.exppara.2016.08.005 27567985

[37] Hai X Zhang H Wang Z Gong H Cao J Zhou Y. Identification of 2-Cys peroxiredoxin (BmTPx-2) as antioxidant active molecule from *Babesia microti*. Front Microbiol. (2017) 8:1959. 10.3389/fmicb.2017.01959 29067017 PMC5641339

[38] Sauri H Butterfield L Kim A Shau H. Antioxidant function of recombinant natural killer enhancing factor. Biochem Biophys Res Commun. (1995) 208:964–9. 10.1006/bbrc.1995.1428 7702627

[39] Thurman RG Ley HG Scholz R. Hydrogen peroxide formation and the role of catalase. Eur J Biochem. (1972) 25:420–30. 10.1111/j.1432-1033.1972.tb01711.x 4402915

[40] Kang SW Baines I Rhee SG. Characterization of a mammalian peroxiredoxin that contains one conserved cysteine. J Biol Chem. (1998) 273:6303–11. 10.1074/jbc.273.11.6303 9497358

[41] Huang J Xiong K Zhang H Zhao Y Cao J Gong H. Molecular characterization of *Babesia microti* thioredoxin (BmTrx2) and its expression patterns induced by antiprotozoal drugs. Parasit Vectors. (2018) 11:38. 10.1186/s13071-018-2619-9 29335000 PMC5769273

[42] Huang J Xiong K Zhang H Zhao Y Cao J Zhou Y. *Babesia microti* thioredoxin 3 is an effective antioxidant and involved in the response to antiprotozoal drugs. Ticks Tick Borne Dis. (2018) 9:645–53. 10.1016/j.ttbdis.2018.01.021 29472160

[43] Pfaffl MW. A new mathematical model for relative quantification in real-time RT-PCR. Nucleic Acids Res. (2001) 29:e45. 10.1093/nar/29.9.e45 11328886 PMC55695

[44] Blasa M Angelino D Gennari L Ninfali P The cellular antioxidant activity in red blood cells (CAA-RBC): a new approach to bioavailability and synergy of phytochemicals and botanical extracts. Food Chem. (2011) 125:685–91. 10.1016/j.foodchem.2010.09.065

[45] Fredriksson K Stridh H Lundahl J Rennard SI Skold CM. Red blood cells inhibit proliferation and stimulate apoptosis in human lung fibroblasts *in vitro*. Scand J Immunol. (2004) 59:559–65. 10.1111/j.1365-3083.2004.01433.x 15182251

[46] Bréhélin C Meyer EH de Souris JP Bonnard G Meyer Y. Resemblance and dissemblance of Arabidopsis type II peroxiredoxins: similar sequences for divergent gene expression, protein localization, and activity. Plant Physiol. (2003) 132:2045–57. 10.1104/pp.103.022533 12913160 PMC181289

[47] Rouhier N Jacquot JP. The plant multigenic family of thiol peroxidases. Free Radic Biol Med. (2005) 38:1413–21. 10.1016/j.freeradbiomed.2004.07.037 15890615

[48] Banerjee M Chakravarty D Ballal A. Redox-dependent chaperone/peroxidase function of 2-Cys-Prx from the cyanobacterium Anabaena PCC7120: role in oxidative stress tolerance. BMC Plant Biol. (2015) 15:60. 10.1186/s12870-015-0444-2 25849452 PMC4349727

[49] Lee EM Lee SS Tripathi BN Jung HS Cao GP Lee Y. Site-directed mutagenesis substituting cysteine for serine in 2-Cys peroxiredoxin (2-Cys Prx A) of Arabidopsis thaliana effectively improves its peroxidase and chaperone functions. Ann Bot. (2015) 116:713–25. 10.1093/aob/mcv094 26141131 PMC4577999

[50] Clarke DJ Ortega XP Mackay CL Valvano MA Govan JR Campopiano DJ. Subdivision of the bacterioferritin comigratory protein family of bacterial peroxiredoxins based on catalytic activity. Biochemistry. (2010) 49:1319–30. 10.1021/bi901703m 20078128

[51] Rhee SG Woo HA Multiple functions of peroxiredoxins: peroxidases, sensors and regulators of the intracellular messenger H_2_O_2_, and protein chaperones. Antioxid Redox Signal. (2011) 15:781–94. 10.1089/ars.2010.3393 20919930

[52] Dietz KJ. Peroxiredoxins in plants and cyanobacteria. Antioxid Redox Signal. (2011) 15:1129–59. 10.1089/ars.2010.3657 21194355 PMC3135184

[53] Okazaki S Naganuma A Kuge S. Peroxiredoxin-mediated redox regulation of the nuclear localization of Yap1, a transcription factor in budding yeast. Antioxid Redox Signal. (2005) 7:327–34. 10.1089/ars.2005.7.327 15706081

[54] Horling F Lamkemeyer P König J Finkemeier I Kandlbinder A Baier M. Divergent light-, ascorbate-, and oxidative stress-dependent regulation of expression of the peroxiredoxin gene family in Arabidopsis. Plant Physiol. (2003) 131:317–25. 10.1104/pp.010017 12529539 PMC166811

[55] Chae HZ Uhm TB Rhee SG. Dimerization of thiol-specific antioxidant and the essential role of cysteine 47. Biochemistry. (1994) 91:7022–26. 10.1073/pnas.91.15.7022 8041739 PMC44330

[56] König J Galliardt H Jütte P Schäper S Dittmann L Dietz KJ. The conformational bases for the two functionalities of 2-cysteine peroxiredoxins as peroxidase and chaperone. J Exp Bot. (2013) 64:3483–97. 10.1093/jxb/ert184 23828546 PMC3733160

[57] Kim YE Hipp MS Bracher A Hayer-Hartl M Hartl FU. Molecular chaperone functions in protein folding and proteostasis. Annu Rev Biochem. (2013) 82:323–55. 10.1146/annurev-biochem-060208-092442 23746257

[58] Cho C Lee GW Hong SH Kaur S Jung KW Jung JH. Novel functions of peroxiredoxin Q from *Deinococcus radiodurans* R1 as peroxidase and a molecular chaperone. FEBS Lett. (2019) 593:219–29. 10.1002/1873-3468.13302 30488429 PMC6590489

[59] Koncarevic S Rohrbach P Deponte M Krohne G Prieto JH Yates J III. The malarial parasite *Plasmodium falciparum* imports the human protein peroxiredoxin 2 for peroxide detoxification. Proc Natl Acad Sci USA. (2009) 106:13323–8. 10.1073/pnas.0905387106 19666612 PMC2726359

[60] Mendes RE Pérez-Ecija RA Zafra R Buffoni L Martínez-Moreno A Dalton JP. Evaluation of hepatic changes and local and systemic immune responses in goats immunized with recombinant Peroxiredoxin (Prx) and challenged with *Fasciola hepatica*. Vaccine. (2010) 28:2832–40. 10.1016/j.vaccine.2010.01.055 20153792

[61] Yano K Komaki-Yasuda K Kobayashi T Takemae H Kita K Kano S. Expression of mRNAs and proteins for peroxiredoxins in *Plasmodium falciparum* erythrocytic stage. Parasitol Int. (2005) 54:35–41. 10.1016/j.parint.2004.08.005 15710548

