# Supplementary figures and images for: A Novel Thioredoxin-Dependent Peroxiredoxin (TPx-Q) Plays an Important Role in Defense Against Oxidative Stress and Is a Possible Drug Target in Babesia microti
Source: Front Vet Sci. 2020 Feb 18;7:76. doi: 10.3389/fvets.2020.00076 (PMC7040034; doi:10.3389/fvets.2020.00076)

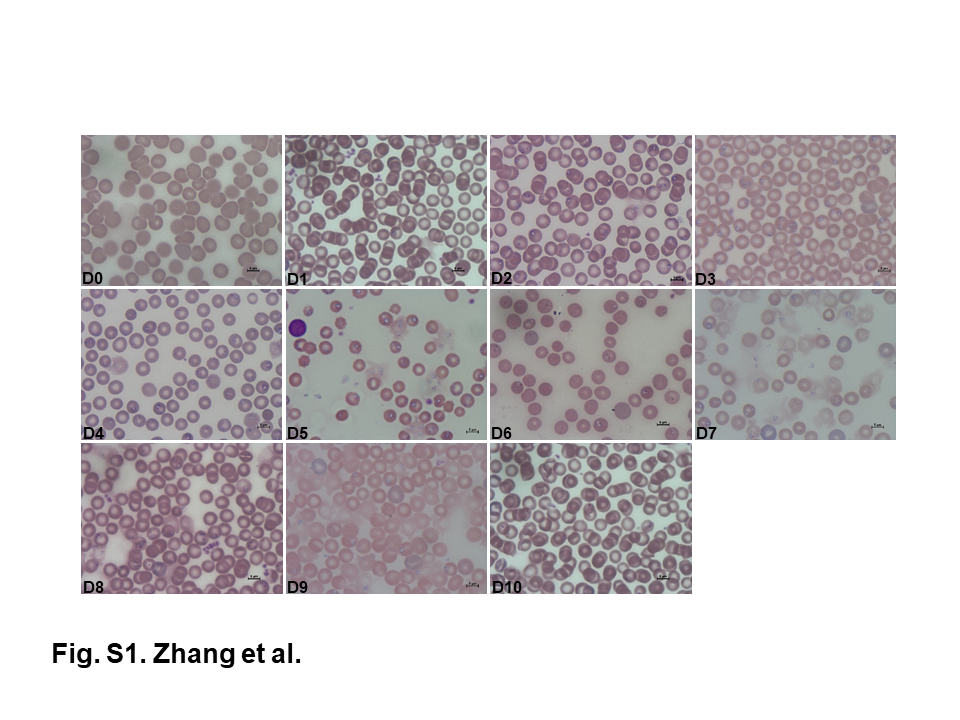

Supplement: Figure S1 — Giemsa-stained thin-blood film of the parasitemia. D0: Uninfected mouse RBCs; D1–D10: Days post-infection with B. microti of mouse RBCs. Scale bar indicates 5.0 μm. [file Image_1.tif]

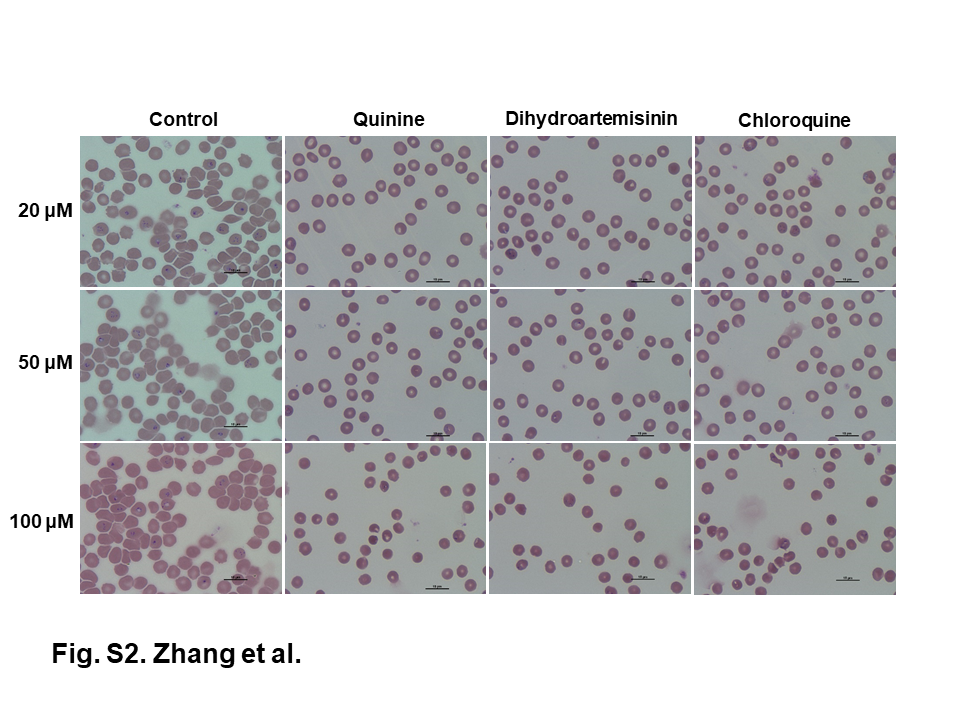

Supplement: Figure S2 — The growth status of B. microti after treatment with antiparasitic agents at 36 h. iRBCs were treated with various concentrations (20, 50, or 100 μM) of Quinine, Dihydroartemisinin, and Chloroquine at 36 h, the controls were treated with DMSO. Thin-blood film was stained with Giemsa. Scale bar indicates 10.0 μm. [file Image_2.tif]
